# Coprophagy Couples Hindgut Fermentation with Multi-Site Microbial Organization in Brandt’s Vole

**DOI:** 10.3390/ani16101514

**Published:** 2026-05-15

**Authors:** Xin-Yi Lu, Xin-Qing Zhao, Sheng-Mei Yang, Wan-Hong Wei, Xin Dai

**Affiliations:** College of Bioscience and Biotechnology, Yangzhou University, 48 East Wenhui Road, Yangzhou 225009, China; luxinyi1423@163.com (X.-Y.L.); zhaoxinqing83@126.com (X.-Q.Z.); smyang@yzu.edu.cn (S.-M.Y.); whwei@yzu.edu.cn (W.-H.W.)

**Keywords:** coprophagy, hindgut fermentation, multi-site microbiota, microbiota structure, Brandt’s vole

## Abstract

Coprophagy, the ingestion of feces, is widespread among vertebrates and plays an important role in hindgut-fermenting herbivores by facilitating nutrient recycling and maintaining gut microbial stability. However, most studies have focused on cecal or fecal microbiota, with limited attention to microbial communities at other body sites. Here, we used Brandt’s vole, a species exhibiting frequent coprophagic behavior, to examine how coprophagy prevention affects hindgut fermentation and microbial communities across multiple sites, including the tongue, lung, stomach, and cecum. Coprophagy prevention increased food intake but reduced body weight gain and significantly decreased cecal acetate, propionate, and butyrate concentrations. At the microbial level, coprophagy prevention increased the Simpson index of the tongue microbiota and induced modest compositional shifts in the tongue, lung, and stomach microbiota. In contrast, the cecal microbiota exhibited greater compositional and predicted functional changes. Notably, coprophagy prevention reduced the Bray–Curtis distance between tongue and cecal microbiota without altering the number of shared amplicon sequence variants. Together, these findings indicate that coprophagy not only promotes efficient nutrient recycling through hindgut fermentation but may also influence microbiota composition across behaviorally interconnected digestive niches.

## Simple Summary

Coprophagy, or the re-ingestion of feces, is a common behavior in many herbivorous mammals and plays an important role in maintaining nutrition and gut function. In this study, we used Brandt’s vole as a model to explore how preventing coprophagy affects digestion and microbial communities throughout the body. When coprophagy was prevented, voles ate more food but gained less weight, and key fermentation products in the cecum (short-chain fatty acids) were significantly reduced. We also found that microbial communities were altered not only in the gut but also in other body sites such as the tongue, lung, and stomach. In particular, the cecal microbiota showed major changes in composition and predicted function. Interestingly, preventing coprophagy made the microbial communities of the tongue and cecum more similar, suggesting that this behavior helps maintain distinct yet coordinated microbial structures across different body sites. Overall, our findings show that coprophagy is not only important for nutrient recycling but also for maintaining the balance and organization of microbial communities throughout the body.

## 1. Introduction

Coprophagy, defined as the ingestion of feces, is a widespread nutritional behavior observed across diverse animal taxa [1,2,3]. This behavior is more common in herbivorous and omnivorous species than in faunivores [4]. In mammals, particularly hindgut-fermenting herbivores, coprophagy enables the recovery of nutrients liberated during microbial fermentation in the hindgut that would otherwise be lost [5]. In small herbivores such as rodents and lagomorphs, coprophagy often involves the selective ingestion of soft, mucus-covered feces originating from the cecum, a process sometimes referred to as cecotrophy [3]. Coprophagy can be further classified as autocoprophagy, the ingestion of an animal’s own feces, or allocoprophagy, the ingestion of feces from conspecifics [6]. Since its first scientific description in rabbits in the late nineteenth century, nutritional coprophagy has been documented in more than 150 vertebrate species, spanning fishes, amphibians, reptiles, birds, and mammals, underscoring its broad evolutionary and ecological significance [4].

In rodents and other small hindgut fermenters, coprophagy is considered an adaptive strategy to overcome metabolic constraints imposed by small body size and fibrous diets [7,8,9]. By re-ingesting microbially enriched feces, animals can enhance the uptake of essential amino acids, vitamins (particularly B-complex vitamins and vitamin K), short-chain fatty acids (SCFAs), and trace elements [10]. Consequently, coprophagy has been associated with improved energy utilization, growth performance, nutritional status, and reproductive outcomes such as pregnancy and lactation [4,5,11,12,13,14,15]. Dietary fiber content has been shown to influence the frequency and physiological importance of coprophagy in herbivorous animals [8,16,17,18,19,20]. Several studies further suggest that coprophagy may contribute to the stabilization of gut microbial communities by facilitating the reintroduction of microbes and their metabolites [5,21,22], thereby promoting microbial metabolism, maintaining host energy balance, and even influencing cognitive performance [5]. Importantly, however, most previous studies on coprophagy in rodents have focused primarily on the cecum or fecal microbiota, with little attention given to microbial communities at other body sites. Whether coprophagy influences microbial colonization and structural relationships beyond the hindgut—such as in the oral cavity, stomach, or respiratory tract—remains poorly understood. Given that coprophagy directly links the oral cavity to the hindgut through repeated ingestion of fecal material, this behavior may play a broader role in shaping multi-site microbial organization and maintaining microbial stability across behaviorally connected niches.

Brandt’s vole (*Lasiopodomys brandtii*) is a small herbivorous rodent inhabiting grassland ecosystems and has been shown to engage in pronounced and rhythmic coprophagic behavior, re-ingesting a substantial proportion of its daily fecal output [7,8]. Previous studies have demonstrated that coprophagy in this species contributes to energy acquisition and growth, stabilizes hindgut microbial communities, promotes microbial metabolism, maintains host energy balance, and influences cognitive performance [5,7,8]. However, the role of coprophagy in regulating microbial communities outside the cecum remains unclear. In the present study, we used Brandt’s vole as a model to investigate how coprophagy prevention influences cecal fermentation and the organization of microbial communities across multiple body sites, including the tongue, lung, stomach, and cecum. By integrating multi-site microbial analyses, we aim to elucidate the role of coprophagy in maintaining energy balance and microbial structure and stability beyond the hindgut, thereby providing new insights into the ecological and physiological functions of this widespread behavioral strategy.

## 2. Materials and Methods

### 2.1. Animals and Treatments

Brandt’s voles used in the experiment were obtained from the laboratory colony maintained by the College of Bioscience and Biotechnology, Yangzhou University, China. The voles, originally captured from the grasslands of Xilinhot, China, were bred under controlled environmental conditions: an ambient temperature of 22 ± 1 °C, a photoperiod of 12 h light/12 h dark (light period: 06:00–18:00), and a relative humidity of 50 ± 5%. All animals had ad libitum access to water and were fed a rodent pellet chow (Yizheng Animal Biotechnology Co., Ltd., Yangzhou, China) containing at least 18% crude protein, no less than 4% crude fat, at most 5% crude fiber, up to 8% ash, and between 1.0% and 1.8% calcium and 0.6% to 1.2% phosphorus. Adult male voles were selected for this study; specifically, at 90 days of age, 30 adult males were randomly chosen and individually housed in polypropylene cages. The selected subjects were acclimated in their respective cages for two weeks prior to the commencement of the experiment. All procedures conformed to the guidelines approved by the Animal Care and Use Committee of the Faculty of Veterinary Medicine at Yangzhou University (protocol number NSFC2020-SKXY-6).

We experimentally inhibited coprophagy in Brandt’s voles by equipping them with a silica gel collar for 15 days. The collars, cut along a radial line and secured around the neck, constituted approximately 5–7% of each vole’s body mass. In the coprophagy prevention (CP) treatment, voles wore a collar 1.5–2 cm in width designed to block access to the anus, thereby preventing fecal consumption. During CP treatments, voles were housed in cages equipped with a wire-mesh floor positioned 2 cm above the base, ensuring that any feces dropping through could not be reached by the mouth. Importantly, these collars did not restrict the voles’ ability to consume chow or drink water. To control for the potential stress of collar wearing, a sham coprophagy prevention (SCP) group was also established. In the SCP group, voles wore a sham-ring collar 0.5 cm in width that allowed them to contact their anus and engage in coprophagy, and the wire mesh was placed directly on the cage floor so that feces remained accessible. In the control group (Con), voles did not wear any collar, and the wire mesh was similarly placed directly on the cage floor. Thirty adult male voles were randomly and equally assigned to three groups. Throughout the experimental period, daily food intake and body weight were recorded. The growth rate of the voles was determined by subtracting the body weight on the first day from that on the last day and then dividing by the initial body weight. Food intake was calculated as the average daily intake over the experimental period. At the conclusion of the experiment, Brandt’s voles were euthanized by rapid decapitation in accordance with institutional animal care guidelines, and samples—including the cecal contents, tongue, lung, and stomach—were collected and stored at −80 °C. The wet weights of the lung and stomach were subsequently measured, and their respective organ indices were calculated.

### 2.2. 16S rRNA Gene Sequencing and Bioinformatic Analysis

To minimize sequencing costs, cecal content, tongue, stomach, and lung tissue samples from eight Brandt’s voles per group, selected using a random-number-based approach, were submitted to Novogene Co., Ltd. (Beijing, China) for 16S rRNA high-throughput sequencing. Sequencing was performed on the Illumina NovaSeq 6000 platform (Illumina, San Diego, CA, USA). The V3–V4 region of the bacterial 16S rRNA gene was amplified using the primers 16SV34-F (CCTAYGGGRBGCASCAG) and 16SV34-R (GGACTACNNGGGTATCTAAT). Raw paired-end reads were merged using FLASH (v1.2.11) and subsequently quality-filtered to generate clean reads. Amplicon sequence variants (ASVs) were identified using the DADA2 pipeline implemented in QIIME2 (version 2022.02). After quality filtering and chimera removal, an average of 77,810 high-quality reads per sample (range: 40,616–152,183) was retained for downstream analyses, corresponding to 78.95% retention of raw reads. Sequencing quality was high, with mean Q30 values exceeding 93.40% (Table S1). To minimize bias caused by uneven sequencing depth, all samples were rarefied to the same sequencing depth corresponding to the sample with the lowest sequence count prior to alpha- and beta-diversity analyses. Rarefaction curves based on observed features approached saturation across all body sites, indicating that the sequencing depth was sufficient to capture the majority of microbial diversity present in the samples (Figure S1). Taxonomic annotation of ASVs was performed against the SILVA database (v138.1) and the NCBI database to determine taxonomic composition and relative abundance profiles. One lung sample from the CP group failed sequencing quality control and was therefore excluded from downstream analyses; consequently, a total of 23 pulmonary tissue samples were included in the final lung microbiota dataset (8 in the NCP group, 8 in the SCP group, and 7 in the CP group). The sequence data have been deposited in the NIH Sequence Read Archive under BioProject accession number PRJNA1394455 (https://dataview.ncbi.nlm.nih.gov/object/PRJNA1394455?reviewer=u20j6j7as87b2lb4een2d0kgfk, accessed on 28 December 2025). Microbial α-diversity indices, including Chao1, observed features, Shannon, Simpson, and Pielou indices, were calculated at the ASV level using QIIME2. Functional prediction of microbial communities was conducted using PICRUSt2 and annotated against the Kyoto Encyclopedia of Genes and Genomes (KEGG) pathway database. According to the sequencing provider, each sequencing batch included matched negative and positive controls that underwent the same amplification, library preparation, sequencing, and bioinformatic processing workflow for quality monitoring and contamination assessment (https://mp.weixin.qq.com/s/24b5jkmnI9Lf1htEbi415A, accessed on 8 March 2026).

### 2.3. SCFA Assay

Cecal SCFA concentrations—including acetate, propionate, butyrate, isobutyrate, valerate, isovalerate, and caproate—were quantified using a gas chromatography–flame ionization detector (GC-FID) following the protocol described by Shen et al. [23]. Samples and standards were analyzed on an Agilent 7080A GC system (Agilent, Santa Clara, CA, USA) equipped with a flame ionization detector (FID) and a Supelco Nukol Fused Silica capillary column (30 m × 0.25 mm, with a film thickness of 0.25 μm). Peak areas were quantified using Agilent Chem Station software (version Rev. B.04.02) (Agilent Technologies, Waldbronn, Germany). The volume of each SCFA in the 0.1 g samples was calculated as the ratio of the peak area of each SCFA in the sample to that of the standards, multiplied by the volume of each SCFA in the 1 mL standards. Finally, the concentration of each SCFA in the cecal samples was determined by dividing the calculated SCFA volume by the mass of the 0.1 g sample.

### 2.4. Statistical Analysis

Body weight changes, daily food intake, organ indices, and cecal SCFA contents were analyzed using one-way analysis of variance (ANOVA) followed by Tukey’s post hoc test after confirming normality using the Shapiro–Wilk test and homogeneity of variances using Levene’s test. Intergroup differences in α-diversity indices were evaluated using nonparametric Kruskal–Wallis tests, with pairwise comparisons adjusted by the Benjamini–Hochberg false discovery rate (FDR) method implemented in the tidyverse R package (v2.0.0). Differential abundances of ASVs and predicted microbial functional pathways (KEGG pathways) were assessed using the MaAsLin2 R package (v1.20.0), with group included as the fixed effect and no additional covariates incorporated into the model. β-diversity of the microbiota was analyzed using permutational multivariate analysis of variance (PERMANOVA) based on Bray–Curtis dissimilarity matrices with the model formula “distance ~ group”, implemented using the adonis2 function in the vegan R package (v2.7.2). Principal coordinate analysis (PCoA) at the ASV level was performed using the ape package (v5.8.1) to visualize microbiota structure, and group separation was further quantified by analysis of similarities (ANOSIM) with 999 permutations. PCoA and ANOSIM visualizations were generated using the ggplot2 R package (v4.0.1). Intergroup differences in Bray–Curtis distances and in the number of shared ASVs between bacterial communities across different body sites were assessed using the vegan and dplyr R packages (v1.1.4) and visualized with ggplot2. Within-group dispersion was evaluated using PERMDISP analysis implemented in the vegan R package. Statistical significance was defined as *p* < 0.05 or *q* < 0.05 (FDR-adjusted *p* value). All ANOVA analyses were performed using IBM SPSS Statistics 26 (IBM, Armonk, NY, USA).

## 3. Results

### 3.1. Effect of Coprophagy Prevention on Food Intake, Growth, and Organ Indices

**Figure 1:**
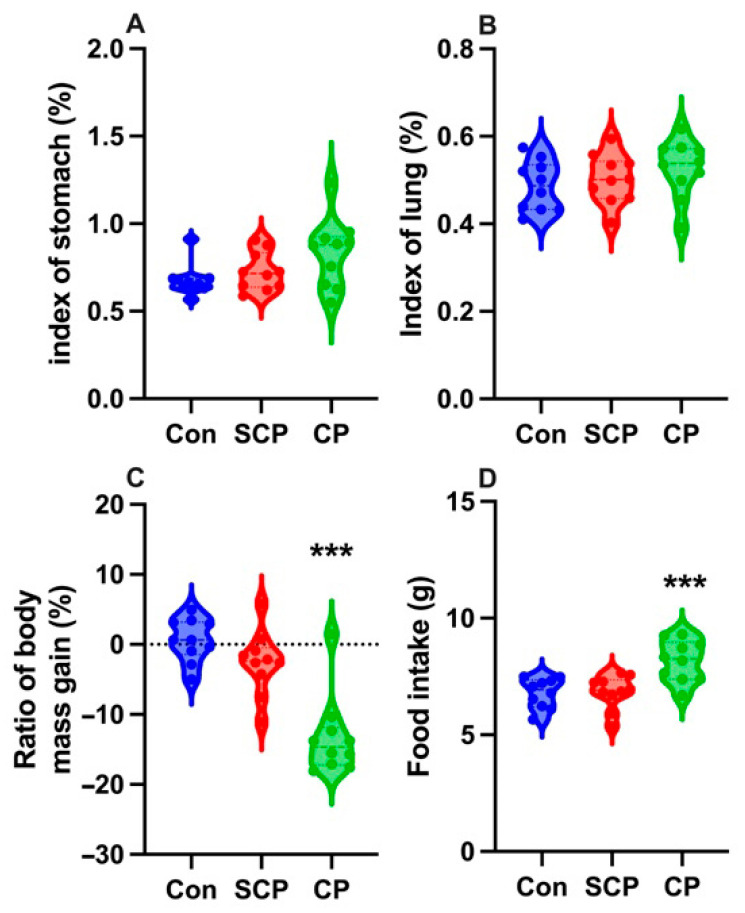
Effects of coprophagy prevention on stomach index (**A**), lung index (**B**), ratio of body mass gain (**C**), and food intake (**D**) in male Brandt’s voles (*n* = 10 per group). Con: control group; SCP: sham coprophagy prevention group; CP: coprophagy prevention group; *** denotes *p* < 0.001 vs. the Con group.

Coprophagy prevention had no significant effect on the stomach or lung indices (stomach: *F*_2,27_ = 3.296, *p* = 0.052; lung: *F*_2,27_ = 1.181, *p* = 0.322) (Figure 1A,B). In contrast, coprophagy prevention significantly affected the ratio of body mass gain (*F*_2,27_ = 25.357, *p* < 0.001) and food intake (*F*_2,27_ = 10.909, *p* < 0.001). Specifically, the ratio of body mass gain (Figure 1C) was significantly lower, and food intake (Figure 1D) was significantly higher in the CP group compared with the control group (both *p* < 0.001). No significant differences were observed between the control and SCP groups for either the ratio of body mass gain (*p* = 0.694) or food intake (*p* = 0.989).

### 3.2. Effect of Coprophagy Prevention on Cecal SCFA Concentrations

**Figure 2:**
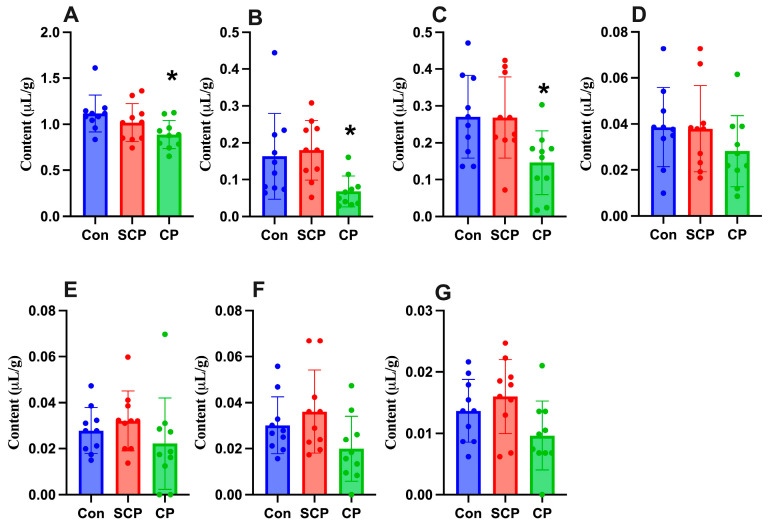
Effects of coprophagy prevention on cecal short-chain fatty acid (SCFA) concentrations in Brandt’s voles: acetate (**A**), propionate (**B**), butyrate (**C**), isobutyrate (**D**), valerate (**E**), isovalerate (**F**), and caproate (**G**) (*n* = 10 per group). Con: control group; SCP: sham coprophagy prevention group; CP: coprophagy prevention group; * denotes *p* < 0.05 vs. the Con group. Data are presented as mean ± SD.

Coprophagy prevention significantly reduced cecal concentrations of acetate (*F*_2,27_ = 3.801, *p* = 0.035), propionate (*F*_2,27_ = 4.988, *p* = 0.014), and butyrate (*F*_2,27_ = 4.719, *p* = 0.017). Compared with the control group, the CP group exhibited significantly lower levels of acetate, propionate, and butyrate (*p* = 0.028, 0.048, and 0.032, respectively), whereas no significant differences were detected between the control and SCP groups (*p* = 0.473, 0.904, and 0.999, respectively; Figure 2A–C). In contrast, CP had no significant effects on the concentrations of isobutyrate, valerate, isovalerate, or caproate (*F*_2,27_ = 1.154, *p* = 0.330; *F*_2,27_ = 1.112, *p* = 0.324; *F*_2,27_ = 2.938, *p* = 0.070; *F*_2,27_ = 3.311, *p* = 0.052, respectively; Figure 2D–G).

### 3.3. Taxonomic Composition of Microbiota Across Body Sites

**Figure 3:**
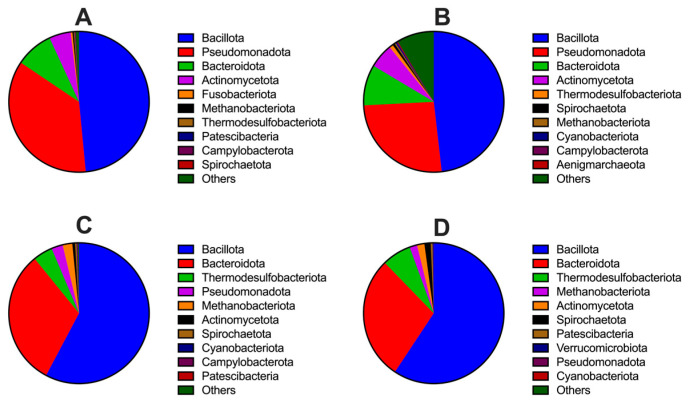
Relative abundance of the top 10 microbial phyla in the tongue (**A**), lung (**B**), stomach (**C**), and cecum (**D**) of Brandt’s voles. Sample sizes were *n* = 24 per site for the tongue, stomach, and cecum microbiota analyses, and *n* = 23 per site for the lung microbiota analysis.

**Figure 4:**
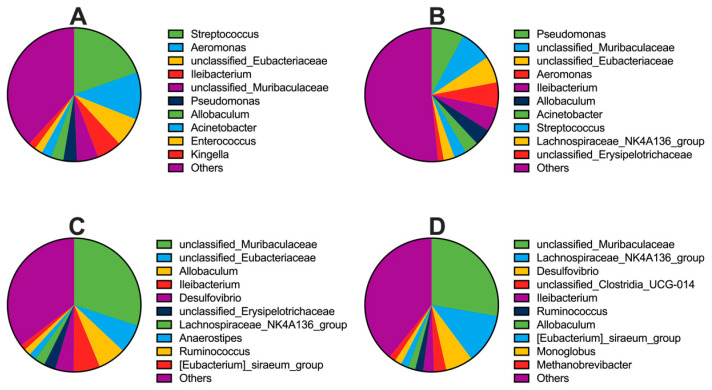
Relative abundance of the top 10 microbial genera in the tongue (**A**), lung (**B**), stomach (**C**), and cecum (**D**) of Brandt’s voles. Sample sizes were *n* = 24 per site for the tongue, stomach, and cecum microbiota analyses, and *n* = 23 per site for the lung microbiota analysis.

Across body sites, distinct microbiota compositions were observed. In the tongue microbiota, the three most abundant phyla were Bacillota (Firmicutes), Pseudomonadota (Proteobacteria), and Bacteroidota (Bacteroidetes) (Figure 3A), and the dominant genera were *Streptococcus*, *Aeromonas*, and unclassified *Eubacteriaceae* (Figure 4A). In the lung microbiota, Bacillota, Pseudomonadota, and Bacteroidota (Figure 3B) were also the dominant phyla, with *Pseudomonas*, unclassified *Muribaculaceae*, and unclassified *Eubacteriaceae* (Figure 4B) as the most abundant genera. In the stomach microbiota, the dominant phyla were Bacillota, Bacteroidota, and Thermodesulfobacteriota (Desulfobacterota) (Figure 3C), and the dominant genera included unclassified *Muribaculaceae*, unclassified *Eubacteriaceae*, and *Allobaculum* (Figure 4C). Similarly, the cecal microbiota was dominated by phyla of Bacillota, Bacteroidota, and Thermodesulfobacteriota (Figure 3D), with unclassified *Muribaculaceae*, *Lachnospiraceae NK4A136 group*, and *Desulfovibrio* as the most abundant genera (Figure 4D).

### 3.4. Effect of Coprophagy Prevention on Alpha Diversity of Microbiota

**Figure 5:**
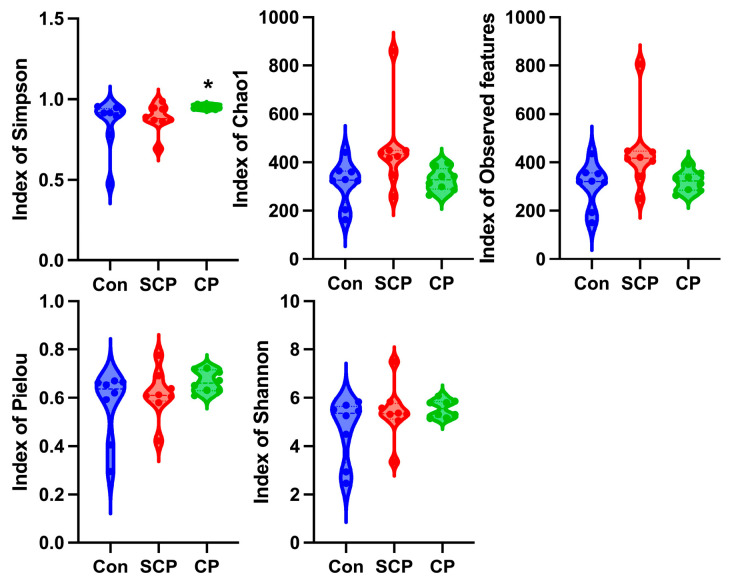
Effect of coprophagy prevention on alpha diversity indices of the tongue microbiota in Brandt’s voles (*n* = 8 per group). Con: control group; SCP: sham coprophagy prevention group; CP: coprophagy prevention group; * denotes *q* < 0.05 vs. the Con group.

In the tongue microbiota, the Simpson index was significantly higher in the CP group than in the control group (*χ*^2^ = 6.622, *p* = 0.036; *q* = 0.047; Figure 3). In contrast, no significant differences were observed between the CP and control groups for Chao1, observed features, Pielou, or Shannon indices (all *q* > 0.05; Figure 5).

No significant differences in alpha-diversity metrics were detected between the CP and control groups in the lung, stomach, or cecal microbiota for observed features, Chao1, Shannon, Pielou, or Simpson indices (all *q* > 0.05; Table S2).

### 3.5. Effect of Coprophagy Prevention on Beta Diversity of Microbiota

PERMANOVA analysis indicated that coprophagy prevention was associated with modest but significant differences in microbiota structure across all four body sites. Significant effects were detected in the tongue (*F* = 1.684, *R*^2^ = 0.138, *p* = 0.021), lung (*F* = 1.350, *R*^2^ = 0.119, *p* = 0.035), stomach (*F* = 1.463, *R*^2^ = 0.122, *p* = 0.016), and cecum (*F* = 1.301, *R*^2^ = 0.110, *p* = 0.044). Pairwise comparisons suggested modest shifts in the tongue (*q* = 0.0795), lung (*q* = 0.054), and stomach (*q* = 0.078) microbiota of the CP group relative to the control group, although these differences reached only marginal significance (*q* < 0.1). In contrast, a significant difference was observed in the cecal microbiota (*q* = 0.021).

**Figure 6:**
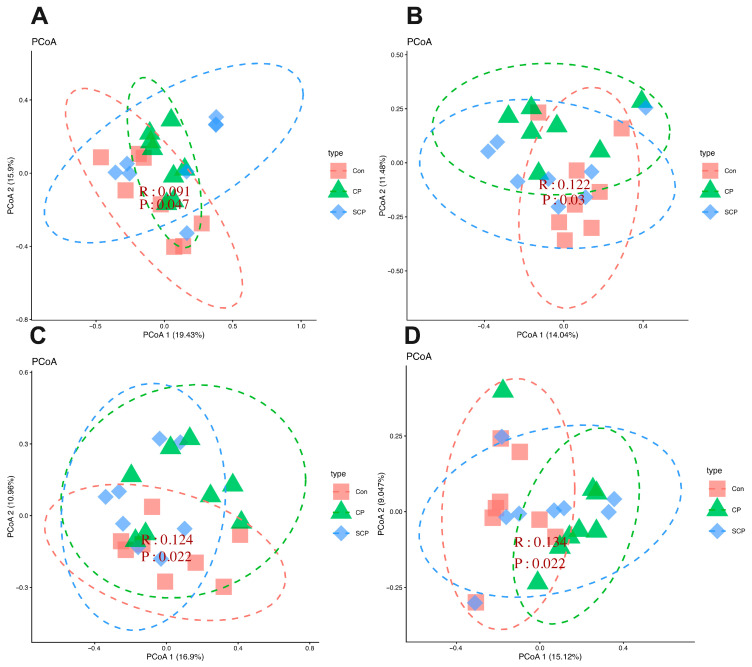
Principal coordinate analysis (PCoA) showing the effects of coprophagy prevention on microbiota structure in the tongue (**A**), lung (**B**), stomach (**C**), and cecum (**D**) of Brandt’s voles. Sample sizes were *n* = 8 per group for the tongue, stomach, and cecum microbiota analyses, and *n* = 8 for the Con and SCP groups and *n* = 7 for the CP group in the lung microbiota analysis. Con, control group; SCP, sham coprophagy prevention group; CP, coprophagy prevention group.

**Figure 7:**
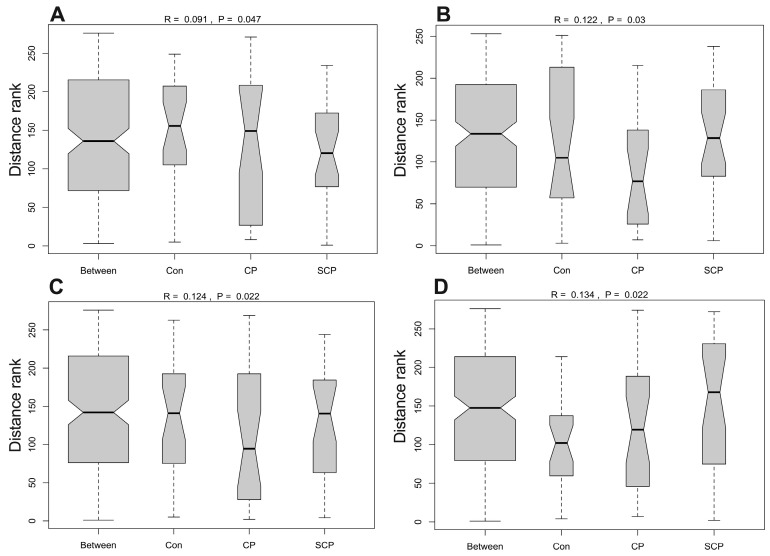
ANOSIM analysis showing the effects of coprophagy prevention on microbiota structure in the tongue (**A**), lung (**B**), stomach (**C**), and cecum (**D**) of Brandt’s voles. Sample sizes were *n* = 8 per group for the tongue, stomach, and cecum microbiota analyses, and *n* = 8 for the Con and SCP groups and *n* = 7 for the CP group in the lung microbiota analysis. Con, control group; SCP, sham coprophagy prevention group; CP, coprophagy prevention group.

PCoA based on Bray–Curtis dissimilarity showed partial separation among groups across all sites (Figure 6A–D). The first two PCoA axes explained 19.43% and 15.90% of the variation in the tongue microbiota, 14.04% and 11.48% in the lung microbiota, 16.90% and 10.96% in the stomach microbiota, and 15.12% and 9.04% in the cecal microbiota. ANOSIM analyses further indicated that intergroup differences exceeded intragroup variation across all sites (*R* = 0.09–0.134, all *p* < 0.05; Figure 7A–D).

PERMDISP analysis showed no significant differences in within-group dispersion between CP and control groups across the tongue, lung, stomach, and cecum microbiota (all *p* > 0.05; Figure S2), indicating that the observed beta-diversity differences were not driven by heterogeneity of dispersion.

### 3.6. Effect of Coprophagy Prevention on Microbial Abundances

**Figure 8:**
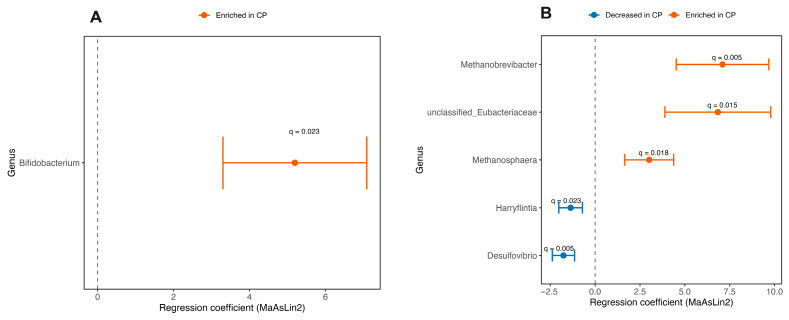
Differentially abundant genera in the lung (**A**) and cecum (**B**) following coprophagy prevention in Brandt’s voles, identified by MaAsLin2 analysis. Sample sizes were *n* = 8 per group for cecum microbiota analyses, and *n* = 8 for the Con group and *n* = 7 for the CP group in the lung microbiota analysis. Con, control group; CP, coprophagy prevention group. Complete MaAsLin2 results are provided in Supplementary Table S3.

MaAsLin2 analysis detected no significant genus-level abundance changes in the tongue or stomach microbiota between the CP and control groups. In the lung microbiota, the relative abundance of *Bifidobacterium* was significantly higher in the CP group than in the control group (*q* = 0.023; Figure 8A). In the cecal microbiota, CP significantly increased the relative abundances of *Methanobrevibacter* (*q* = 0.005), unclassified *Eubacteriaceae* (*q* = 0.015), and *Methanosphaera* (*q* = 0.018), while significantly decreasing the relative abundances of *Desulfovibrio* (*q* = 0.005) and *Harryflintia* (*q* = 0.023; Figure 8B).

### 3.7. Effect of Coprophagy Prevention on Predicted Cecal Microbial Functions

**Figure 9:**
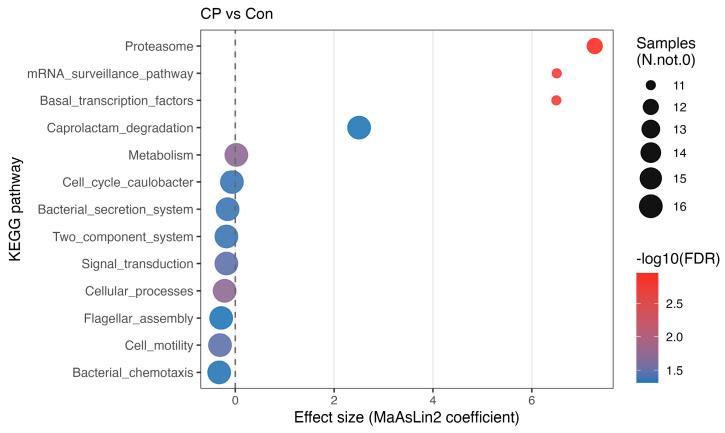
Differential KEGG pathways in the cecal microbiota following coprophagy prevention in Brandt’s voles, identified by MaAsLin2 analysis (*n* = 8 per group). Con, control group; CP, coprophagy prevention group. Complete MaAsLin2 results are provided in Supplementary Table S4.

At KEGG pathway level 1, coprophagy prevention increased the relative abundance of pathways associated with metabolism (*q* = 0.019) and decreased those associated with cellular processes (*q* = 0.019). At level 2, pathways related to cell motility and signal transduction were significantly reduced in the CP group (both *q* = 0.034). At level 3, pathways including proteasome, basal transcription factors, mRNA surveillance, and caprolactam degradation were enriched in the CP group, whereas pathways associated with bacterial secretion systems, two-component systems, chemotaxis, and flagellar assembly were significantly depleted (all *q* < 0.05; Figure 9).

### 3.8. Effect of Coprophagy Prevention on Inter-Site Microbial Distance and Shared Taxa

**Figure 10:**
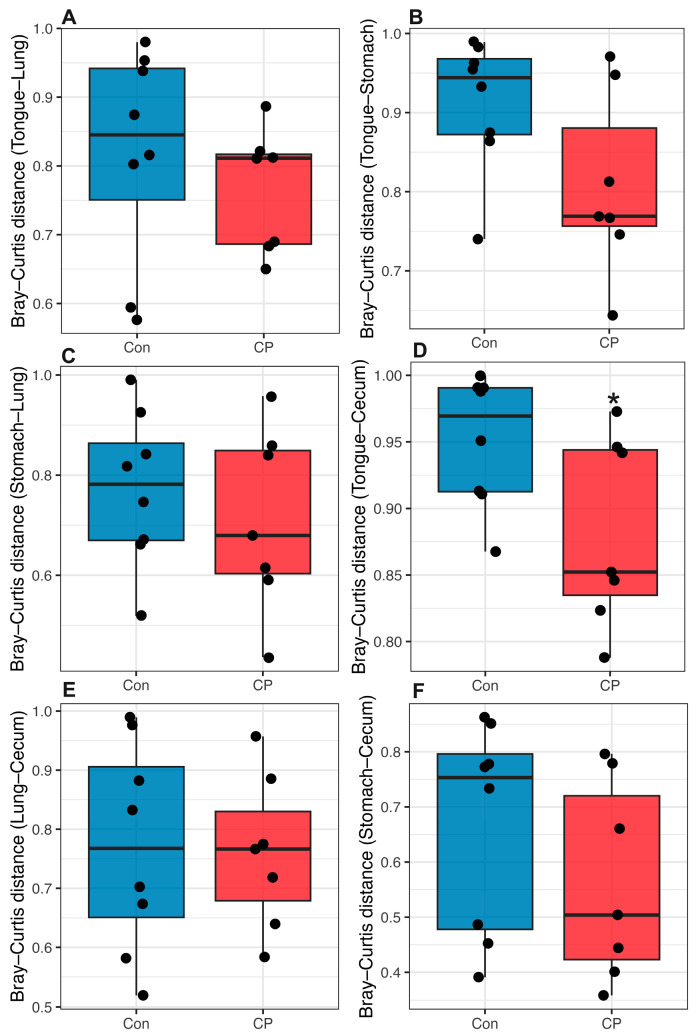
Effects of coprophagy prevention on Bray–Curtis dissimilarity between microbial communities from different anatomical sites in Brandt’s voles. Lower Bray–Curtis distances indicate greater compositional similarity between microbial communities. Pairwise comparisons between the control (Con) and coprophagy prevention (CP) groups were performed for tongue–lung (**A**), tongue–stomach (**B**), stomach–lung (**C**), tongue–cecum (**D**), lung–cecum (**E**), and stomach–cecum (**F**) microbial communities. Coprophagy prevention significantly reduced the Bray–Curtis distance between tongue and cecal microbiota, indicating increased compositional similarity between these two sites. Sample sizes were *n* = 8 for the Con group and *n* = 7 for the CP group. * *p* < 0.05 versus the Con group.

The Bray–Curtis distance between tongue and cecal microbiota was significantly lower in the CP group than in the control group (*W* = 46, *p* = 0.040; Figure 10). In contrast, no significant differences were observed for pairwise distances among other body sites (all *p* > 0.05; Figure 10).

**Figure 11:**
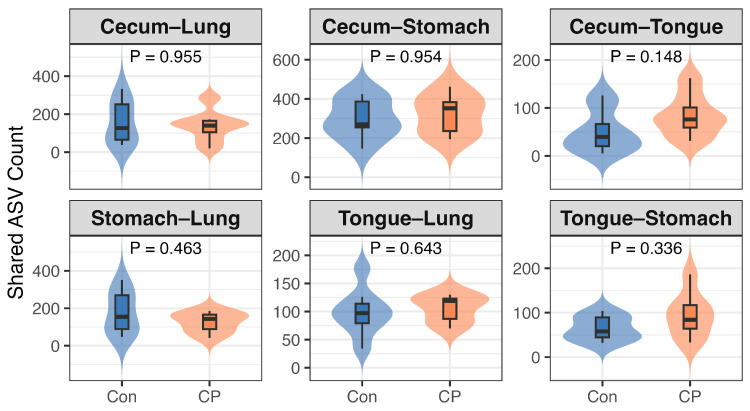
Effects of coprophagy prevention on inter-site microbial ASV sharing in Brandt’s voles. Sample sizes were *n* = 8 per group for the Con group, and *n* = 7 per group for the CP group. Con, control group; CP, coprophagy prevention group.

Similarly, the number of shared ASVs between any pair of body sites did not differ significantly between the CP and control groups (all *p* > 0.05), although the largest difference was observed for shared ASVs between the tongue and cecum (*p* = 0.148; Figure 11).

## 4. Discussion

Our study demonstrates that coprophagy prevention leads to a clear mismatch between energy intake and growth performance in Brandt’s vole. Despite a marked increase in food intake, coprophagy prevention significantly reduced body mass gain and cecal concentrations of acetate, propionate, and butyrate, indicating impaired hindgut fermentation and inefficient nutrient utilization. Consistent with our findings, Bo et al. [5] also reported reduced body mass accompanied by increased food consumption following coprophagy prevention in adult male Brandt’s voles, reinforcing the notion that coprophagy is essential for maintaining energy balance rather than simply supplementing caloric intake. Coprophagy in rodents involves the ingestion of soft feces rich in microbially derived nutrients, including vitamins, amino acids, and peptides [8], and its restriction has been shown to induce signs of malnutrition, such as shortened intestinal villi [5]. Accordingly, the increased food intake observed in the present study failed to compensate for the loss of body mass gain, suggesting that coprophagy prevention deprives voles of critical nutrients or microbial metabolites that cannot be fully replaced by additional feed consumption. Although previous work proposed that coprophagy prevention limits access to bacteria-derived nutrients, particularly short-chain fatty acids [5], the specific nutritional components responsible for the observed body mass loss remain unresolved in our study and warrant further investigation.

Consistent with the impaired growth performance observed following coprophagy prevention, the concentrations of acetate, propionate, and butyrate in the cecum were markedly reduced, a pattern also reported in Brandt’s voles [5]. These short-chain fatty acids (SCFAs) are the primary end products of anaerobic microbial fermentation of dietary fiber in the hindgut and constitute a major energy source for the host [24]. After absorption, acetate, propionate, and butyrate contribute to glucose, lipid, and cholesterol metabolism and act as important metabolic substrates beyond the gut [25,26]. In addition, SCFAs function as signaling molecules that influence systemic energy homeostasis and regulate appetite through the stimulation of gut-derived satiety hormones [25,27]. In this context, the reduced SCFA concentrations observed in the present study indicate impaired microbial fermentation efficiency, which likely limited metabolizable energy availability and contributed to attenuated body weight gain despite increased food intake. Such an uncoupling suggests that the elevated food consumption represented a compensatory response to reduced fermentative energy yield rather than enhanced nutritional efficiency. This finding further underscores the importance of SCFA-mediated hindgut fermentation in maintaining host energy balance.

In the cecal microbiota, alpha-diversity metrics did not change significantly following coprophagy prevention, whereas beta-diversity analyses indicated modest but significant differences in community structure. A similar pattern was reported in the fecal microbiota of adult male Brandt’s voles, where alpha-diversity indices such as the Shannon index and observed OTUs remained unchanged, while beta diversity differed significantly after coprophagy prevention [5]. Alpha diversity generally reflects species richness and evenness within a microbiota, whereas beta diversity assesses compositional dissimilarity between microbial communities based on sequence abundance or presence–absence information [28,29,30]. Together, these findings suggest that coprophagy prevention does not substantially affect overall microbial richness or evenness but instead induces compositional reorganization through changes in the abundance of specific taxa. Consistently, several genera with differential abundance were identified by MaAsLin2 analysis in the present study.

*Methanobrevibacter* uses H_2_ and/or formate to reduce CO_2_ to CH_4_ [31,32,33] and aids in digesting complex polysaccharides by optimizing hydrogen levels [34]. *Methanosphaera*, common in herbivores [35], produces CH_4_ from methanol with H_2_ [36,37]. Their increased levels suggest enhanced CH_4_ production and H_2_ elimination in the cecum. Since H_2_ and formate are key fermentation byproducts [38], methanogens help prevent their accumulation and sustain fermentation efficiency. Although lower SCFA levels indicate reduced fermentative activity, the enrichment of these methanogens may improve fermentation under metabolic stress. Meanwhile, *Desulfovibrio*—a hydrogen sulfide producer using hydrogen, SCFAs, and other substrates [39]—declines due to competition for hydrogen and reduced SCFA availability. Similarly, decreased *Harryflintia* aligns with lower acetate levels [40,41], while increased unclassified *Eubacteriaceae* may help maintain SCFA production by fermenting dietary fiber to butyrate [42]. Overall, the reduction in SCFA levels after coprophagy prevention likely reflects a reorganization of SCFA-producing taxa, not a complete fermentation shutdown.

In the study by Bo et al. [5], coprophagy prevention significantly reduced the relative abundance of several genera, including *Oscillospira*, *Rikenella*, *Anaerostipes*, *Oxalobacter*, and *Clostridium*, while *Prevotella* and *Dorea* increased. In contrast, none of these genera exhibited significant abundance changes in the present study. At the phylum level, Bo et al. [5] further reported a decrease in Bacillota and an increase in Bacteroidota after two weeks of coprophagy prevention, whereas neither phylum showed significant changes in our study, despite being the two dominant phyla in the cecal microbiota in both studies. Bacillota and Bacteroidetes have also been consistently reported as the dominant gut phyla in laboratory Brandt’s voles [43,44]. Dietary fiber is known to shape gut microbial composition and fermentative capacity in the host [45,46,47]. The gut microbiota of Brandt’s voles varies with factors such as age, sex, diet, and environment [44]. In this context, the difference in dietary fiber content between studies may contribute to the divergent microbial responses to coprophagy prevention. The chow used in the present study contained approximately 5% crude fiber, whereas the diet used by Bo et al. [5] contained ~12% crude fiber, indicating a substantially higher proportion of poorly digestible plant structural components in the latter. Consistent with this, the relative abundance of Bacillota in the control group reported by Bo et al. [5] was slightly higher than that observed in our study (approximately 70% vs. 59%). Diets with higher fiber content are therefore likely to impose a greater dependence on cellulose-degrading bacteria and microbial fermentation, thereby amplifying the impact of coprophagy prevention on gut microbial composition. It is therefore possible that the relatively low-fiber diet used in the present study attenuated the magnitude of microbial responses to coprophagy prevention compared with studies using higher-fiber diets. Despite the relatively low-fiber diet, significant changes in SCFA concentrations, body weight gain, and microbiota structure were still detected, suggesting that coprophagy prevention exerted measurable biological effects under the present experimental conditions. Overall, these findings suggest that the effects of coprophagy prevention on the gut microbiota are context-dependent, lacking uniform taxonomic responses and being strongly modulated by dietary composition and other environmental factors.

Previous studies have associated pathways related to bacterial cell motility, such as flagellar assembly, with microbial metabolic characteristics in mammals [48,49,50,51,52]. In addition, the two-component system pathway is generally involved in microbial environmental sensing and signal-response regulation [53,54,55]. In the present study, the relative abundance of these predicted pathways was reduced in the CP group and coincided with decreased body weight gain. However, because KEGG annotations generated by PICRUSt2 represent inferred functional potential rather than direct functional measurements, these findings should be interpreted cautiously. The observed pathway shifts may reflect altered predicted functional profiles of the cecal microbiota rather than confirmed changes in microbial metabolic activity or nutrient-sensing processes. Similarly, although metabolism-related pathways appeared to increase overall, this pattern was largely driven by caprolactam degradation, a pathway categorized under xenobiotic biodegradation and metabolism [56], and therefore should not be interpreted as direct evidence of enhanced energy-harvesting efficiency or fermentation activity.

Bacillota, Pseudomonadota, and Bacteroidota dominated the tongue microbiota, echoing studies in rodents and humans reporting Bacillota, Pseudomonadota, and Bacteroidota [57,58,59]. The lung showed a similar profile, aligning with reports of pulmonary communities enriched in these phyla plus Actinobacteria [57,60]. In the stomach and cecum, Bacillota and Bacteroidota were predominant, consistent with findings in mice and Brandt’s voles [5,61,62], with Thermodesulfobacteriota also notable in the cecum [63,64]. These findings support the reliability of the sequencing data from all sites.

An increased Simpson index indicates reduced dominance of highly abundant taxa and greater community evenness [30,65]. Thus, the elevated Simpson index observed in the tongue microbiota after coprophagy prevention suggests a more even distribution of bacterial taxa rather than increased microbial richness. Because no individual genera showed significant differential abundance in the MaAsLin2 analysis, these findings likely reflect modest compositional shifts distributed across multiple taxa rather than significant changes in specific bacterial groups.

However, alpha-diversity metrics for the lung and stomach microbiota did not show significant differences between groups, suggesting that preventing coprophagy did not substantially influence within-sample richness or evenness at these sites. Conversely, beta-diversity analyses indicated slight differences in community composition between groups (*p* < 0.1), which is more reflective of an emerging trend toward community reorganization rather than a complete restructuring. This pattern, along with the unchanged alpha diversity across most sites and consistent within-group dispersion, implies that coprophagy prevention led to modest shifts in microbiota composition without significantly altering overall diversity or community stability. Although coprophagy prevention was associated with reduced cecal SCFA concentrations and impaired growth performance, MaAsLin2 analysis revealed a higher relative abundance of Bifidobacterium in the lung microbiota of the CP group. Bifidobacterium is commonly regarded as a commensal genus with potential immunomodulatory properties [66,67]. However, because the lung represents a low-biomass microbial environment that is highly susceptible to host filtering effects and potential technical contamination [68,69], the biological significance of this finding should not be overinterpreted. Rather than indicating improved host health, the observed increase may reflect subtle shifts in microbial composition associated with physiological stress or altered host conditions following coprophagy prevention.

Inter-site beta-diversity analysis showed that preventing coprophagy markedly diminished the Bray–Curtis distance between cecal and tongue microbiotas, while pairwise distances among other anatomical sites and the shared ASV count remained constant. The unchanged number of shared ASVs suggests that the reduced tongue–cecum dissimilarity was unlikely to result from increased microbial exchange or direct taxonomic convergence. Instead, these findings may reflect parallel shifts in microbial community composition across both sites. Considering that coprophagy contributes to nutrient recycling, hindgut fermentation, and microbial metabolite production, its interruption may have contributed to changes in the physiological environment of both the oral cavity and the cecum simultaneously. In particular, the reduction in cecal short-chain fatty acids observed in the present study may have influenced mucosal immune tone, epithelial homeostasis, and nutrient availability, thereby potentially affecting microbiota composition at both locations. Coprophagy prevention may also induce broader metabolic or inflammatory changes that could contribute to greater compositional similarity between anatomically connected digestive niches. However, because the number of shared ASVs remained unchanged, the observed reduction in Bray–Curtis distance is more likely to reflect altered relative abundance patterns rather than increased microbial transfer between sites. Additional analyses, such as source-tracking, microbial co-occurrence analysis, or network-based approaches, will be necessary to further clarify the ecological relationships underlying these inter-site compositional changes. From an ecological perspective, coprophagy has long been regarded as an adaptive nutritional strategy in hindgut-fermenting herbivores because it facilitates the recovery of microbially derived nutrients and supports digestive efficiency under fiber-rich dietary conditions [4,5,70,71]. Increasing evidence further suggests that coprophagic behavior may contribute to the stabilization of host-associated microbiota by repeatedly reintroducing gut-derived microbes into the digestive system [72]. In this context, the present findings further support the idea that coprophagy can influence host-associated microbial ecology and potentially affect host energy utilization as well as microbiota composition across interconnected digestive niches.

This study presents several limitations that merit consideration. First, although preventing coprophagy was associated with lower SCFA levels and reduced weight gain, the specific nutrients that were absent due to this intervention remain unidentified. Second, variations in diet and experimental conditions may affect the observed responses, thereby limiting the generalizability of our findings. In addition, although the SCP group was designed to control for the potential effects of collar wearing, differences in collar width, feces accessibility, and wire-mesh floor configuration between the CP and SCP groups may have introduced additional environmental or physical influences that could potentially affect host physiology and microbiota composition independent of coprophagy prevention. Therefore, the observed microbial and physiological alterations warrant cautious interpretation in terms of direct causality specifically attributable to coprophagy prevention alone. One lung sample from the CP group failed sequencing quality control and was excluded from downstream analyses, resulting in a slightly reduced sample size for lung microbiota analyses. Given the relatively high inter-individual variability commonly observed in microbiome datasets, this reduction may have decreased the statistical power for detecting subtle differences, particularly in beta-diversity analyses. Therefore, several microbiota differences that reached only marginal significance (*q* < 0.1) should be interpreted conservatively until validated in larger cohorts. Nevertheless, the overall microbiota patterns remained generally consistent across analyses. Furthermore, although batch-level negative and positive controls were incorporated by the sequencing provider during sequencing and data processing, dedicated contaminant-filtering approaches (e.g., Decontam) were not independently applied in the present study. Therefore, the potential influence of low-biomass or reagent-derived contamination, particularly in lung microbiota analyses, cannot be completely excluded. Future studies incorporating dedicated contaminant-filtering pipelines will help further validate the biological significance of low-abundance microbial taxa. Finally, while there was a strong association between coprophagy, microbiota composition, and host energy balance, additional mechanistic studies integrating mucosal immunity, metabolomics, source-tracking approaches, and microbial interaction analyses will be necessary to clarify the ecological relationships underlying the observed compositional shifts across digestive tract niches.

## 5. Conclusions

This study demonstrates that coprophagy plays an important role in linking hindgut fermentation with microbiota organization in Brandt’s voles. Preventing coprophagy impaired nutrient recycling efficiency, as evidenced by reduced short-chain fatty acid concentrations and attenuated body weight gain despite increased food intake. At the microbial level, coprophagy prevention exerted the strongest effects on the cecal microbiota, while comparatively modest compositional shifts were observed in the extracecal microbiota. Notably, the reduced Bray–Curtis distance between tongue and cecal microbiota occurred without increased microbial sharing, suggesting altered relative abundance patterns rather than direct microbial translocation between sites. Collectively, these findings suggest that coprophagy may contribute to the maintenance of microbiota organization across behaviorally interconnected digestive niches, thereby linking digestive efficiency with microbiota composition in multiple body sites. Consequently, disruption of this behavior may influence host energy balance and microbiota structure across interconnected digestive regions.

## Data Availability

The microbial sequence data are available at the NIH Sequence Read Archive with Bioproject ID PRJNA1394455.

## Supplementary Materials

The following supporting information can be downloaded at: https://www.mdpi.com/article/10.3390/ani16101514/s1, Figure S1. Observed features rarefaction curves of the tongue, lung, stomach and cecum microbiota (*n* =24 per site). The uppermost curve represents the lung sample from the CP group that failed sequencing quality control. Figure S2. Within-group dispersion of microbiota between the coprophagy prevention (CP), sham coprophagy prevention (SCP), and control (Con) groups across the tongue (A), lung (B), stomach (C), and cecum (D). Sample sizes were *n* = 8 per group for the tongue, stomach, and cecum microbiota analyses, and *n* = 8 for the Con and SCP groups and *n* = 7 for the CP group in the lung microbiota analysis. Table S1. Summary of sequencing depth and quality control metrics for all samples. Table S2. Alpha-diversity parameters of microbiota in the lung, stomach, and cecum of Brandt’s voles (Mean ± SEM). Table S3. MaAsLin2 results for differentially abundant genera in the cecal and pulmonary microbiota. Table S4. MaAsLin2 results for differentially abundant predicted KEGG pathways in the cecal microbiota (*n* = 8 per group).
