# Supplementary material for: Coprophagy Couples Hindgut Fermentation with Multi-Site Microbial Organization in Brandt’s Vole
Source: Animals (Basel). 2026 May 15;16(10):1514. doi: 10.3390/ani16101514 (PMC13203151; doi:10.3390/ani16101514)
Supplement: Supplementary file 1 [file animals-16-01514-s001.zip › animals-4283915-supplementary.pdf]

Supplementary Figures

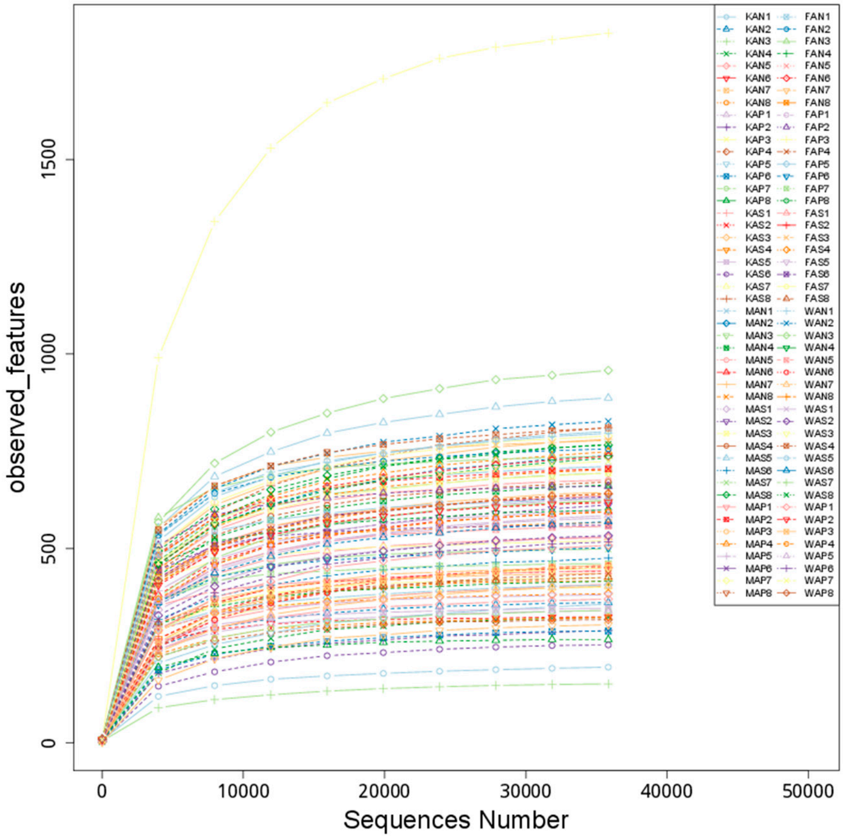

Figure S1. Observed features rarefaction curves of the tongue, lung, stomach and cecum microbiota (*n* =24 per site). The uppermost curve represents the lung sample from the CP group that failed sequencing quality control.

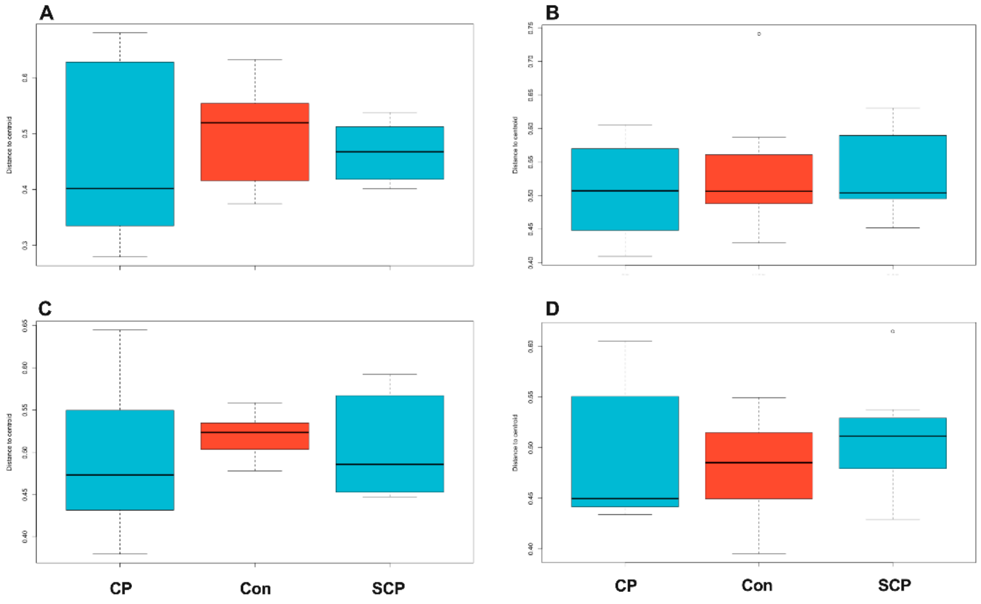

Figure S2. Within-group dispersion of microbiota between the coprophagy prevention (CP), sham coprophagy prevention (SCP), and control (Con) groups across the tongue (A), lung (B), stomach (C), and cecum (D). Sample sizes were *n* = 8 per group for the tongue, stomach, and cecum microbiota analyses, and *n* = 8 for the Con and SCP groups and *n* = 7 for the CP group in the lung microbiota analysis.

Supplementary Tables

Supplementary Table S1. Summary of sequencing depth and quality control metrics for all samples.

Sample	Site	Group	RawPE	Qualified	Nochime	Q30
FAN1	Lung	NCP	121864	112600	103068	93.65%
FAN2	Lung	NCP	107318	103823	90255	96.07%
FAN3	Lung	NCP	106960	102962	89126	95.80%

FAN4	Lung	NCP	102309	98435	90027	95.98%
FAN5	Lung	NCP	77560	74582	69848	96.48%
FAN6	Lung	NCP	101952	77008	74175	93.80%
FAN7	Lung	NCP	102796	99298	93717	94.68%
FAN8	Lung	NCP	105635	99592	80509	96.32%
FAP1	Lung	CP	169923	139027	131899	94.07%
FAP2	Lung	CP	103907	98578	79886	96.10%
FAP4	Lung	CP	113034	109921	99614	96.18%
FAP5	Lung	CP	82802	72475	71106	93.40%
FAP6	Lung	CP	100632	97866	91468	96.60%
FAP7	Lung	CP	105807	103154	83817	96.21%
FAP8	Lung	CP	78910	68545	61170	94.74%
FAS1	Lung	SCP	81457	79792	67846	96.47%
FAS2	Lung	SCP	102543	100424	53220	95.30%
FAS3	Lung	SCP	105171	103197	87414	96.24%
FAS4	Lung	SCP	96390	81455	78609	93.91%
FAS5	Lung	SCP	105386	101866	63942	95.85%
FAS6	Lung	SCP	53508	51849	42208	96.21%
FAS7	Lung	SCP	104077	99463	91504	96.30%
FAS8	Lung	SCP	81858	76692	75111	96.15%
KAN1	Tongue	NCP	102548	99457	75347	95.69%
KAN2	Tongue	NCP	106447	103726	60029	94.96%
KAN3	Tongue	NCP	89372	86941	62627	94.70%
KAN4	Tongue	NCP	94994	92116	87084	94.67%
KAN5	Tongue	NCP	162154	156235	152183	95.33%

KAN6	Tongue	NCP	70512	68807	64310	95.43%
KAN7	Tongue	NCP	57507	55730	44423	94.98%
KAN8	Tongue	NCP	87823	84952	73298	95.28%
KAP1	Tongue	CP	105662	102498	57905	95.35%
KAP2	Tongue	CP	106082	103399	66718	95.30%
KAP3	Tongue	CP	106608	103538	83929	95.50%
KAP4	Tongue	CP	85411	82938	51719	95.02%
KAP5	Tongue	CP	91125	88348	79424	95.17%
KAP6	Tongue	CP	73201	71392	54767	95.24%
KAP7	Tongue	CP	112068	109100	73065	95.07%
KAP8	Tongue	CP	67515	65736	50065	95.14%
KAS1	Tongue	SCP	80489	78519	69648	95.95%
KAS2	Tongue	SCP	86769	84526	79270	95.84%
KAS3	Tongue	SCP	57221	55631	49762	96.00%
KAS4	Tongue	SCP	52208	50942	40616	95.07%
KAS5	Tongue	SCP	75880	73636	66257	95.27%
KAS6	Tongue	SCP	103576	100384	87592	94.69%
KAS7	Tongue	SCP	67893	66317	41757	94.90%
KAS8	Tongue	SCP	105964	103312	75320	95.57%
MAN1	Cecum	NCP	77442	74785	53173	94.51%
MAN2	Cecum	NCP	105910	101756	77733	93.90%
MAN3	Cecum	NCP	105288	101672	66051	94.52%
MAN4	Cecum	NCP	105617	101593	85687	94.24%
MAN5	Cecum	NCP	100117	96674	82861	94.48%
MAN6	Cecum	NCP	104197	100310	77983	93.95%

MAN7	Cecum	NCP	102408	98748	77148	94.25%
MAN8	Cecum	NCP	86494	83744	73760	94.68%
MAP1	Cecum	CP	104458	100646	73753	94.10%
MAP2	Cecum	CP	97432	93871	71011	94.49%
MAP3	Cecum	CP	101470	97983	71658	94.37%
MAP4	Cecum	CP	103082	99526	80239	94.54%
MAP5	Cecum	CP	102791	99272	77252	94.78%
MAP6	Cecum	CP	106388	102641	90048	94.66%
MAP7	Cecum	CP	102733	99091	78576	94.64%
MAP8	Cecum	CP	106736	103005	83496	94.57%
MAS1	Cecum	SCP	94918	91921	73645	94.74%
MAS2	Cecum	SCP	104701	100970	74096	94.51%
MAS3	Cecum	SCP	103738	100500	64521	94.59%
MAS4	Cecum	SCP	102151	98680	74455	94.46%
MAS5	Cecum	SCP	119204	114764	86062	94.21%
MAS6	Cecum	SCP	107334	103756	81826	94.59%
MAS7	Cecum	SCP	55902	54198	43303	95.14%
MAS8	Cecum	SCP	106048	102765	85113	94.89%
WAN1	Stomach	NCP	101015	97542	75720	94.34%
WAN2	Stomach	NCP	105417	101347	80764	94.01%
WAN3	Stomach	NCP	104472	100297	83086	94.13%
WAN4	Stomach	NCP	103662	100048	86238	94.12%
WAN5	Stomach	NCP	82196	79051	76210	94.26%
WAN6	Stomach	NCP	102714	98427	88092	93.65%
WAN7	Stomach	NCP	105052	100918	93085	94.12%

WAN8	Stomach	NCP	103623	100195	89740	94.21%
WAP1	Stomach	CP	103669	100025	94679	94.56%
WAP2	Stomach	CP	106192	102507	85311	94.31%
WAP3	Stomach	CP	81457	78587	70608	94.43%
WAP4	Stomach	CP	109255	105559	94135	94.67%
WAP5	Stomach	CP	105368	101669	84378	94.36%
WAP6	Stomach	CP	106237	102493	95361	94.46%
WAP7	Stomach	CP	103550	100108	83021	94.71%
WAP8	Stomach	CP	104492	100781	81486	94.30%
WAS1	Stomach	SCP	105350	102135	85959	94.51%
WAS2	Stomach	SCP	118621	114544	93498	94.56%
WAS3	Stomach	SCP	105120	101822	88689	94.40%
WAS4	Stomach	SCP	103298	99890	75478	94.40%
WAS5	Stomach	SCP	103968	100089	80774	93.90%
WAS6	Stomach	SCP	102342	98975	89661	94.45%
WAS7	Stomach	SCP	103231	100011	90351	94.93%
WAS8	Stomach	SCP	119214	115458	104599	94.71%

Note: Sample sizes were *n* = 8 per group for the tongue, stomach, and cecum microbiota analyses, and *n* = 8 for the Con and SCP groups and *n* = 7 for the CP group in the lung microbiota analysis.

Supplementary Table S2. Alpha-diversity parameters of microbiota in the lung, stomach, and cecum of Brandt's voles (Mean ± SEM).

Body position	Parameters of alpha diversity	Con	SCP	CP
Lung	Chao1	591.43±62.56	513.62±47.15	545.60±38.51

	Observed features	573.63±60.41	503.63±45.78	527.71±38.23
	Pielou	0.71±0.08	0.72±0.03	0.73±0.04
	Shannon	6.54±0.75	6.46±0.31	6.60±0.39
	Simpson	0.89±0.09	0.96±0.01	0.95±0.02
Stomach	Chao1	707.37±73.17	672.74±52.00	606.44±33.21
	Observed features	671.25±67.90	641.878±47.69	580.50±30.00
	Pielou	0.70±0.03	0.74±0.02	0.69±0.03
	Shannon	6.61±0.41	6.85±0.19	6.29±0.34
	Simpson	0.96±0.01	0.98±0.004	0.94±0.02
Cecum	Chao1	727.93±22.83	667.78±53.08	701.26±25.62
	Observed features	695.13±19.38	641.63±50.18	672.13±23.94
	Pielou	0.76±0.01	0.74±0.01	0.75±0.01
	Shannon	7.16±0.13	6.86±0.19	7.05±0.14
	Simpson	0.98±0.004	0.97±0.004	0.98±0.004

Con: Control group; SCP: Sham coprophagy prevention group; CP: Coprophagy prevention group. Sample sizes were n = 8 per group for stomach, and cecum microbiota analyses, and n = 8 for the Con and SCP groups and n = 7 for the CP group in the lung microbiota analysis.

Supplementary Table S3. MaAsLin2 results for differentially abundant genera in the cecal and pulmonary microbiota.

Site	Feature (genus)	metadata	value	coef	stderr	N	N.not.0	pval	qval
Cecum	Desulfovibrio	group	CP	-1.7654404	0.31652931	16	16	6.81E-05	0.00478138
	Methanobrevibacter	group	CP	7.09777306	1.31593774	16	8	9.47E-05	0.00478138
	unclassified_Eubacteriaceae	group	CP	6.83801382	1.50457708	16	10	0.00045822	0.01542661
	Methanosphaera	group	CP	3.00944986	0.69690455	16	11	0.00070803	0.01787765
	Harryflintia	group	CP	-1.3663383	0.33603508	16	6	0.00115635	0.02335822
Lung	Bifidobacterium	group	CP	5.1938213	0.96570896	15	13	0.00012574	0.02275958

Note: Sample sizes were $n = 8$ per group for the cecum microbiota analyses, and $n = 8$ for the Con and SCP groups and $n = 7$ for the CP group in the lung microbiota analysis.

Supplementary Table S4. MaAsLin2 results for differentially abundant predicted KEGG pathways in the cecal microbiota ($n = 8$ per group).

Variable-KEGG	Group1	Group2	W_statistic	p_value	BH_adjusted_p	Significance
Proteasome	NCP	CP	64	0.00086	0.00258	Yes
Basal.transcription.factors	NCP	CP	63	0.00115	0.00345	Yes
mRNA.surveillance.pathway	NCP	CP	63	0.00115	0.00345	Yes
Bacterial.secretion.system	NCP	CP	1	0.00136	0.00408	Yes
Caprolactam.degradation	NCP	CP	60	0.00388	0.01163	Yes
Signal.transduction	NCP	CP	4	0.00388	0.01163	Yes
Two.component.system	NCP	CP	4	0.00388	0.01163	Yes
Phosphotransferase.system..PTS.	SCP	CP	60	0.00388	0.01163	Yes
Carbon.fixation.by.Calvin.cycle	NCP	CP	59	0.00538	0.01615	Yes
Cell.cycle...Caulobacter	NCP	CP	5	0.00538	0.01615	Yes
Cellular.Processes	NCP	CP	5	0.00538	0.01615	Yes

Flagellar.assembly	NCP	CP	5	0.00538	0.01615	Yes
Glycolysis...Gluconeogenesis	SCP	CP	59	0.00538	0.01615	Yes
Cell.motility	NCP	CP	6	0.00741	0.02222	Yes
Metabolism	NCP	CP	58	0.00741	0.02222	Yes
Sulfur.metabolism	NCP	CP	6	0.00741	0.02222	Yes
Folding..sorting.and.degradation	NCP	CP	9	0.01813	0.02719	Yes
Carbon.fixation.by.Calvin.cycle	SCP	CP	55	0.01813	0.02719	Yes
Folding..sorting.and.degradation	SCP	CP	8	0.01359	0.02719	Yes
Bacterial.chemotaxis	NCP	CP	7	0.01008	0.03025	Yes
Cell.growth.and.death	NCP	CP	7	0.01008	0.03025	Yes
Fatty.acid.biosynthesis	NCP	CP	7	0.01008	0.03025	Yes
Lipoic.acid.metabolism	NCP	SCP	57	0.01008	0.03025	Yes
Protein.export	NCP	CP	8	0.01359	0.04076	Yes
Selenocompound.metabolism	NCP	CP	8	0.01359	0.04076	Yes
Amino.sugar.and.nucleotide.sugar.metabolism	SCP	CP	56	0.01359	0.04076	Yes
Glycerophospholipid.metabolism	NCP	SCP	56	0.01359	0.04076	Yes
Arginine.and.proline.metabolism	NCP	CP	54	0.02395	0.04699	Yes
C5.Branched.dibasic.acid.metabolism	NCP	CP	11	0.03132	0.04699	Yes
Glycerophospholipid.metabolism	NCP	CP	11	0.03132	0.04699	Yes
Sulfur.relay.system	NCP	CP	9	0.01813	0.04699	Yes
Arginine.and.proline.metabolism	SCP	CP	53	0.03132	0.04699	Yes
C5.Branched.dibasic.acid.metabolism	SCP	CP	9	0.01813	0.04699	Yes
Sulfur.relay.system	SCP	CP	11	0.03132	0.04699	Yes
Cell.cycle...Caulobacter	NCP	SCP	53	0.03132	0.04699	Yes
Digestive.system	NCP	CP	55	0.01813	0.05439	Yes

Environmental.adaptation	NCP	CP	9	0.01813	0.05439	Yes
Plant.pathogen.interaction	NCP	CP	9	0.01813	0.05439	Yes
Protein.digestion.and.absorption	NCP	CP	55	0.01813	0.05439	Yes
Sphingolipid.metabolism	NCP	CP	55	0.01813	0.05439	Yes
Ascorbate.and.aldarate.metabolism	SCP	CP	55	0.01813	0.05439	Yes
Penicillin.and.cephalosporin.biosynthesis	SCP	CP	55	0.01813	0.05439	Yes
Proteasome	NCP	SCP	12	0.03764	0.05647	Yes
Bacterial.chemotaxis	SCP	CP	12	0.04057	0.06085	Yes
Sphingolipid.metabolism	SCP	CP	52	0.04057	0.06085	Yes
Steroid.biosynthesis	NCP	SCP	10	0.02191	0.06573	Yes
Human.Diseases	NCP	CP	51	0.05203	0.07804	Yes
Other.glycan.degradation	NCP	CP	51	0.05203	0.07804	Yes
Cell.motility	SCP	CP	13	0.05203	0.07804	Yes
Cellular.Processes	SCP	CP	13	0.05203	0.07804	Yes
Other.glycan.degradation	SCP	CP	52	0.04057	0.07804	Yes
Cell.growth.and.death	NCP	SCP	51	0.05203	0.07804	Yes
Human.Diseases	NCP	SCP	11	0.03132	0.07804	Yes
Immune.system	SCP	CP	10.5	0.02731	0.08193	Yes
NOD.like.receptor.signaling.pathway	SCP	CP	10.5	0.02731	0.08193	Yes
Ribosome.biogenesis.in.eukaryotes	NCP	CP	53	0.03132	0.09397	Yes
Alanine..aspartate.and.glutamate.metabolism	SCP	CP	53	0.03132	0.09397	Yes
Galactose.metabolism	SCP	CP	53	0.03132	0.09397	Yes
Benzoate.degradation	NCP	SCP	11	0.03132	0.09397	Yes
Tyrosine.metabolism	NCP	SCP	53	0.03132	0.09397	Yes
Flagellar.assembly	SCP	CP	14	0.06608	0.09912	Yes

Metabolism	SCP	CP	50	0.06608	0.09912	Yes
Signal.transduction	SCP	CP	14	0.06608	0.09912	Yes
Two.component.system	SCP	CP	14	0.06608	0.09912	Yes
Amino.sugar.and.nucleotide.sugar.metabolism	NCP	SCP	50	0.06608	0.09912	Yes

Note: The pathways presented in Figure 9 are highlighted in red.
